# Which patient and surgeon characteristics are associated with surgeon experience of stress during an office visit?^☆^

**DOI:** 10.1016/j.pecinn.2022.100043

**Published:** 2022-04-22

**Authors:** Tom Crijns, Aresh Al Salman, Laura Bashour, David Ring, Teun Teunis, The Science of Variation Group

**Affiliations:** a Dell Medical School, The University of Texas at Austin, Austin, TX, USA; b University of Pittsburgh Medical Center, Pittsburgh, PA, USA

**Keywords:** Clinician experience, Stress level, Futility, Practice variation, Job satisfaction

## Abstract

**Objective:**

To determine clinician and patient factors associated with the surgeon feelings of stress, futility, inadequacy, and frustration during an office visit.

**Methods:**

A survey-based experiment presented clinical vignettes with randomized patient factors (such as symptom intensity, the number of prior consultations, and involvement in a legal dispute) and feeling behind schedule in order to determine which are most related to surgeon ratings of stress, futility, inadequacy, and frustration on 11-point Likert scales.

**Results:**

Higher surgeon stress levels were independently associated with women patients, multiple prior consultations, a legal dispute, disproportionate symptom intensity, and being an hour behind in the office. The findings were similar for feelings of futility, inadequacy, and frustration.

**Conclusion:**

Patient factors potentially indicative of mental and social health opportunities are associated with greater surgeon-rated stress and frustration.

**Innovation:**

Trainings for surgeon self-awareness and effective communication can transform stressful or adversarial interactions into an effective part of helping patients get and stay healthy by diagnosing and addressing psychosocial aspects of the illness.

**Level of evidence:**

N/a

## Highlights

• Surgeon-rated feelings of stress in office visit scenarios are associated with multiple specialist evaluation, disproportionate symptoms, and ongoing legal dispute.
• Many surgeons desired additional training in effective communication strategies, discernment of the psychosocial aspects of illness, assistance with them in the office, and more effective planning and strategizing for patient encounters.
• The association between factors that reflect a mismatch between patient and specialist symptom interpretation with physician stress suggests that surgeon stress may be an indicator of patient mental and social health opportunities.
• Immersion in the biopsychosocial paradigm and improved coordination of care with social workers or clinical psychologists may help limit surgeon stress when encountering patients with notable psychosocial aspects of illness.

## 1 Introduction

Adversarial patient-clinician interactions—where patient and clinician have different interpretations of the symptoms and different priorities—can be demoralizing. Adversarial interactions are relatively common (15% in a study of non-specialty care [1]) and are associated with greater symptom intensity, higher utilization of care, and lower satisfaction, all of which are associated with opportunities for surgeons to address low mental health such as unhelpful thinking regarding symptoms or feelings of worry or despair regarding symptoms [1,2]. There is evidence that adversarial patient-clinician relationships hinder diagnosis of pathology [3,4], and that patient-clinician discordance is an indication of mental and social health opportunities (room for patients to improve security in life roles, finances, housing, food, etc. and for physicians to help or direct care) which can benefit from diagnosis and treatment [[5], [6], [7]]. Physician attitudes (e.g. stigmatization of mental health, discomfort with uncertainty), non-technical skills (e.g. communication strategies), and behaviors (e.g. blaming others, difficulty accepting failure, and perfectionism) may contribute to conflict with patients [2,[8], [9], [10], [11]], while effective communication strategies and effective patient-clinician relationships can bolster joy in practice [12].

There is a gap in evidence regarding aspects of patient encounters that surgeons find frustrating and stressful, how they interpret those interactions, and what actions surgeons take as a result. It is not clear that surgeons have sufficient tools and training for effective relationships with patients who express different perspectives. A better understanding of surgeon and patient factors associated with surgeon-rated discomfort in patient interactions has the potential to increase self-awareness and aid quality improvement efforts.

In a cross-sectional survey-based experiment of a large group of international surgeons we asked: 1) Are there clinician and patient factors independently associated with the surgeon’s rating a visit as stressful?; 2) Are there any factors associated with surgeon-rated feelings of: futility, inadequacy, or frustration?; 3) What is the daily proportion of patient encounters that feel adversarial?; and 4) What options, tools, or additional training do surgeons feel might limit adversarial relationships with patients?

## 2 Materials and methods

### 2.1 Study design and setting

This study was approved by our Institutional Review Board. We distributed a survey-based experiment (SurveyMonkey, Palo Alto, CA) to all members of the Science of Variation Group (SOVG) in December 2020. The SOVG is an international consortium founded by one of the senior authors of this manuscript that has more than 750 orthopedic, plastic, and general trauma surgeons who are interested in variation in care and complete monthly surveys on a voluntary basis. Participants receive no financial incentives. Survey links were distributed by email with 2 weekly reminders. Completing the survey represents consent. All participants were asked to complete clinical vignettes with randomized patient and practice variables as presented in Table 1; we used simple randomization for each variable. Surgeons were randomized to complete either 3 or 5 clinical vignettes (simple randomization, 1:1) to balance achieving sufficient statistical power while mitigating questionnaire fatigue.

**Table 1:** Clinical vignettes.

Variable	Options	Sentence			
Age/Gender	1. A	A 30-year-old man seeks your care for the first time			
	B	A 60-year-old man seeks your care for the first time			
	C	A 30-year-old woman seeks your care for the first time			
	D	A 60-year-old woman seeks your care for the first time			
Prior consultations	2. A	The patient has consulted with 3 other specialists. The patient was frustrated by those encounters and didn’t feel taken seriously.			
	B	The patient has consulted with another specialist. The patient wants to discuss the encounters and compare your opinion.			
	C	(nothing)			
Pre-visit investigation	3. A	The patient has done some investigating on the internet and has some ideas about what’s going on and what to do.			
	B	The patient has done some investigating on the internet and feels confused, looking to you for guidance.			
	C	(nothing)			
Secondary gain	4. A	The symptoms arose after an event at work and the patient has felt unable to work since.			
	B	The symptoms arose after an accident and the patient has an ongoing lawsuit against the person involved.			
	C	(nothing)			
Symptom intensity	5. A	The patient describes unbearable pain and finds it difficult to complete simple activities of daily living.			
	B	The patient is able to maintain their daily routine.			
Available time	6. A	You are nearly an hour behind in the office.			
	B	It’s a light day and you are not feeling rushed.			

Each item was randomized through simple randomization (1:4, 1:3, or 1:2).

### 2.2 Measured variables

We collected the following information about participating surgeons: surgeon gender, practice location, years in practice, subspecialty, and supervision of trainees. Each vignette included the following variables: patient age and gender, prior consultations by another specialist, pre-visit internet searches, secondary gain (benefit) from illness, symptom intensity, and available time in the office. These variables were chosen based on group discussion of factors that might potentially be a cause of clinician stress. For each scenario, surgeons were asked to imagine treating this patient and to rate their 1) level of stress and feelings of 2) futility, 3) inadequacy, and 4) frustration on a 11-point Likert scale (ranging from 0, minimum, to 10, maximum) [[13], [14], [15]]. Additionally, surgeons were invited to suggest, after each vignette, tools or trainings that might help them better navigate these encounters. Finally, surgeons were asked what proportion of patient encounters they experience as challenging on a daily basis.

### 2.3 Participants

A total of 111 surgeons completed the survey-based experiment. Most surgeons were men (94%, n=97), practicing in the United States (50%, n=51) or Europe (35%, n=36). The majority was sub-specialized in hand or wrist surgery (40%, n=41), or trauma surgery (31%, n=32), and the majority supervised trainees (82%, n=84) (Table 2). The experiments largely rely on diversity of responses within the sample and are therefore less influenced by participation rate, which is not possible to measure with our experimental design. The number of participants and their practice and demographic characteristics were comparable to previous studies by our collaboration [[16], [17], [18], [19]].

**Table 2:** Surgeon demographics

Variable	Value
N	111
Gender	
Men	97 (94%)
Women	6 (5.8%)
Practice location	
United States	51 (50%)
Europe	36 (35%)
Other	16 (16%)
Years in practice	
0 to 5	26 (25%)
6 to 10	21 (20%)
11 to 20	31 (30%)
21 to 30	25 (24%)
Subspecialty	
Hand and wrist	41 (40%)
Trauma	32 (31%)
Shoulder and elbow	14 (14%)
Other	16 (16%)
Supervising trainees	
Yes	84 (82%)
No	19 (18%)
Emotions (11-point ordinal scale)	
Stress	5 (2-6)
Futility	5 (3-6)
Inadequacy	2 (1-5)
Frustration	4 (2-7)

Discrete variables as number (percentage); continuous variables as median (interquartile range). Eight surgeons had missing data.

### 2.4 Statistical analysis

Discrete variables were presented as number (percentage) and continuous variables as median (interquartile range). We decided apriori to address patient factors using a multi-level model given that there is intercorrelation (nesting) on a surgeon-level that should be taken into account given that each surgeon completed multiple cases. In addition, we constructed a (single level) multivariable model to seek surgeon factors associated with our response variables. Because patient factors are presented in a randomized fashion, this model does not have to account for patient factors.

#### 2.4.1 Patient variables

Because we expected that each patient variable would have varying effects on each surgeon surveyed, and cases were correlated within observers (each surgeon completed 3 or 5 cases), we decided to perform multi-level modelling, nesting cases within each surgeon to assess the effect of patient variables. Each multi-level model yielded a statistically significant likelihood-ratio test compared to a linear model, indicating that the random effects model provides better fit. We did not include surgeon characteristics in the random-effects equation for simplicity and because it did not yield a notable difference in patient-level estimates, which were deemed more important. Four separate multi-level mixed-effects linear regression models were constructed, seeking factors associated with 1) stress, 2) futility, 3) inadequacy, and 4) frustration, accounting for patient age, gender, the number of consultations by another specialist, the extent of pre-visit investigation, presence (and type of) secondary gain from illness, symptom intensity, and available time in the office.

#### 2.4.2 Surgeon variables

We created 4 multivariable linear regression models with the mean 1) stress, 2) futility, 3) inadequacy, and 4) frustration level of each surgeon as the dependent variable. In these models we accounted for surgeon gender, practice location, years in practice, subspecialty, and supervision of trainees.

Finally, a multivariable negative binomial regression model was constructed, to identify surgeon factors associated with the proportion of difficult cases in daily practice as rated by surgeons.

To indicate the magnitude of effect, the Akaike Information Criterion (AIC) of the full model was compared to the model in the absence of each of the variables (delta-AIC). Lower AIC values indicate better fit, and therefore, higher delta-AIC values indicate that a larger proportion of variation in the dependent variable is explained by the factor [20]. Regression Coefficients, 95% Confidence Intervals, and *P*-values, and delta-AIC values were reported.

An a priori sample size calculation determined that 136 rated vignettes would provide 80% power to detect statistical significance based on a multivariable linear regression model with alpha set at 0.05, if (assumption 1 [[16], [17], [18], [19]]) a variable explained at least 9.5% of the variation in surgeon stress level, and if (assumption 2 [[16], [17], [18], [19]]) the full model explained 15% or more of the total variation. Because rated vignettes are correlated (nested) within observers, we assumed a correlation of 0.50 and doubled our sample size, resulting in 272 ratings in total, equating to 68 participants (given that surgeons rate 4 cases on average [3 or 5 vignettes; simple randomization]).

## 3 Results

Accounting for potential confounding in multilevel mixed-effects linear regression analysis, higher surgeon stress levels were independently associated with women patients, prior consultation with 3 other specialists, an ongoing lawsuit or Workers’ Compensation claim, disproportionate symptom intensity, and being an hour behind in the office (Table 3). Being behind in the office explained the largest proportion of variation in stress levels (highest delta-AIC). In linear regression analysis, lower surgeon stress levels were associated with practicing in Europe and the subspecialty ‘Shoulder and elbow’ (Table 4); the latter explaining the greatest proportion of variation.

**Table 3:** Multilevel mixed-effects linear regression analysis of patient factors associated with the clinician experience, accounting for nesting by surgeons.

	Stress			Futility		
Variables	Regression coefficient (95% Confidence Interval)	Δ AIC*	*P* value	Regression coefficient (95% Confidence Interval)	Δ AIC*	*P* value
Age (reference: 30)						
60	-0.083 (-0.39 to 0.22)	-1.7	0.592	-0.062 (-0.41 to 0.29)	-1.9	0.728
Gender (reference: man)						
Woman	0.41 (0.111 to 0.71)	5.1	**0.007**	0.31 (-0.040 to 0.66)	1.0	0.083
Prior consultations (reference: none)						
One	0.084 (-0.30 to 0.47)	14	0.668	-0.11 (-0.56 to 0.33)	21	0.616
Three	0.73 (0.36 to 1.1)		**<0.001**	0.91 (0.48 to 1.3)		**<0.001**
Pre-visit investigation (reference: none)						
Some	-0.044 (-0.43 to 0.34)	-2.6	0.824	-0.24 (-0.68 to 0.20)	-2.7	0.282
Extensive	0.17 (-0.20 to 0.54)		0.369	-0.054 (-0.49 to 0.38)		0.807
Secondary gain (reference: none)						
Workers' Compensation	0.63 (0.26 to 0.99)	17	**0.001**	0.45 (0.028 to 0.87)	2.2	**0.037**
Ongoing lawsuit	0.89 (0.50 to 1.3)		**<0.001**	0.52 (0.066 to 0.97)		**0.025**
Symptom intensity (reference: expected)						
Disproportionate	0.39 (0.076 to 0.70)	3.9	**0.015**	0.44 (0.078 to 0.80)	3.6	**0.017**
Available time (reference: plenty)						
Little	2.1 (1.8 to 2.4)	141	**<0.001**	0.58 (0.22 to 0.93)	8.0	**0.001**


	Inadequacy			Frustration		
Variables	Regression coefficient (95% Confidence Interval)	Δ AIC*	*P* value	Regression coefficient (95% Confidence Interval)	Δ AIC*	*P* value
Age (reference: 30)						
60	-0.048 (-0.33 to 0.23)	-1.9	0.735	-0.085 (-0.40 to 0.23)	-1.7	0.598
Gender (reference: man)						
Woman	0.25 (-0.024 to 0.53)	1.2	0.073	0.38 (0.066 to 0.69)	3.6	**0.018**
Prior consultations (reference: none)						
One	-0.030 (-0.38 to 0.32)	8.2	0.869	-0.24 (-0.64 to 0.16)	22	0.234
Three	0.51 (0.17 to 0.86)		**0.003**	0.74 (0.36 to 1.1)		**<0.001**
Pre-visit investigation (reference: none)						
Some	-0.081 (-0.43 to 0.27)	1.0	0.653	0.081 (-0.32 to 0.48)	2.7	0.690
Extensive	0.30 (-0.046 to 0.64)		0.090	0.48 (0.096 to 0.87)		**0.015**
Secondary gain (reference: none)						
Workers' Compensation	0.13 (-0.20 to 0.47)	6.0	0.431	0.41 (0.029 to 0.79)	15	**0.035**
Ongoing lawsuit	0.56 (0.20 to 0.92)		**0.002**	0.91 (0.50 to 1.3)		**<0.001**
Symptom intensity (reference: expected)						
Disproportionate	0.35 (0.069 to 0.64)	3.8	**0.015**	0.51 (0.19 to 0.84)	7.5	**0.002**
Available time (reference: plenty)						
Little	0.29 (0.010 to 0.57)	2.1	**0.042**	1.3 (0.94 to 1.6)	52	**<0.001**

**Bold** indicates statistical significance, *P* < 0.05. *Δ AIC = Akaike Information Criterion; AIC of the full model compared to model without each variable. Higher values indicate better fit.

**Table 4:** Multivariable linear regression analysis of surgeon factors associated with the clinician experience.

	Stress			Futility		
Variables	Regression coefficient (95% Confidence Interval)	Δ AIC*	*P* value	Regression coefficient (95% Confidence Interval)	Δ AIC*	*P* value
Gender (reference: woman)						
Man	-0.045 (-1.2 to 1.2)	-2.0	0.941	0.34 (-0.81 to 1.5)	-1.6	0.557
Practice location (reference: United States)						
Europe	-1.0 (-1.7 to -0.37)	5.8	**0.002**	-0.46 (-1.1 to 0.17)	-1.8	0.153
Other	-0.50 (-1.3 to 0.26)		0.197	-0.094 (-0.83 to 0.64)		0.801
Years in practice (reference: 0 to 5)						
6 to 10	0.70 (-0.074 to 1.5)	-0.6	0.076	0.11 (-0.64 to 0.85)	2.3	0.776
11 to 20	0.40 (-0.32 to 1.1)		0.276	0.088 (-0.60 to 0.78)		0.802
21 to 30	-0.11 (-0.89 to 0.66)		0.771	-0.80 (-1.5 to -0.063)		**0.034**
Subspecialty (reference: hand and wrist)						
Shoulder and elbow	-1.8 (-2.6 to -0.96)	14	**<0.001**	-1.5 (-2.3 to -0.75)	9.4	**<0.001**
Trauma	-0.31 (-0.99 to 0.35)		0.352	-0.47 (-1.1 to 0.18)		0.155
Other	-0.14 (-0.96 to 0.69)		0.745	-0.20 (-0.99 to 0.60)		0.629
Supervising trainees (reference: no)						
Yes	-0.18 (-0.88 to 0.52)	-1.7	0.614	-0.34 (-1.0 to 0.33)	-1.0	0.321


	Inadequacy			Frustration		
Variables	Regression coefficient (95% Confidence Interval)	Δ AIC*	*P* value	Regression coefficient (95% Confidence Interval)	Δ AIC*	*P* value
Gender (reference: woman)						
Man	1.1 (-0.0036 to 2.2)	1.9	0.051	0.11 (-1.1 to 1.3)	-2.0	0.855
Practice location (reference: United States)						
Europe	0.19 (-0.40 to 0.78)	4.5	0.524	-1.1 (-1.7 to -0.40)	6.2	**0.002**
Other	1.0 (0.31 to 1.7)		**0.004**	-0.49 (-1.3 to 0.28)		0.214
Years in practice (reference: 0 to 5)						
6 to 10	0.53 (-0.17 to 1.2)	18	0.140	0.55 (-0.24 to 1.3)	12	0.170
11 to 20	0.17 (-0.48 to 0.82)		0.605	0.72 (-0.0063 to 1.5)		0.052
21 to 30	-1.1 (-1.8 to -0.44)		**0.001**	-0.75 (-1.5 to 0.037)		0.062
Subspecialty (reference: hand and wrist)						
Shoulder and elbow	-1.7 (-2.4 to -0.98)	17	**<0.001**	-1.7 (-2.5 to -0.92)	14	**<0.001**
Trauma	-0.70 (-1.3 to -0.092)		**0.024**	-0.54 (-1.2 to 0.14)		0.117
Other	-0.035 (-0.78 to 0.71)		0.926	0.14 (-0.70 to 0.97)		0.748
Supervising trainees (reference: no)						
Yes	-0.42 (-1.0 to 0.21)	-0.26	0.194	-0.66 (-1.4 to 0.048)	1.4	0.068

**Bold** indicates statistical significance, *P* < 0.05. *Δ AIC = Akaike Information Criterion; AIC of the full model compared to model without each variable. Higher values indicate better fit.

In multilevel mixed-effects linear regression analysis, greater surgeon-rated feelings of futility were associated with prior consultations with 3 specialists, an ongoing lawsuit or Workers’ Compensation claim, disproportionate symptom intensity, and being an hour behind in the office. Greater feelings of inadequacy were associated with prior consultations with 3 specialists, an ongoing lawsuit, disproportionate symptoms, and being behind an hour behind in the office. Greater surgeon-rated frustration was associated with women patients, prior consultations with 3 specialists, extensive pre-visit internet searches, an ongoing lawsuit or Workers’ Compensation claim, disproportionate symptoms, and less available time. Surgeons with the subspecialty ‘Shoulder and elbow’ felt less futility, inadequacy, and frustration; and trauma surgeons felt less inadequate. Surgeons with more than 20 years of experience felt less futility and inadequacy. European surgeons reported lower levels of frustration, while surgeons not practicing in the US or Europe felt more inadequate.

The median percentage of challenging encounters reported by surgeons was 20%, with an interquartile range of 15% to 31%. In multivariable negative binomial regression analysis, a lower surgeon-rated proportion of challenging encounters was associated with practicing in the US and the subspecialties ‘Shoulder and elbow’ and ‘trauma’ (Table 5).

**Table 5:** Multivariable negative binomial regression analysis of factors associated with the proportion of difficult cases in daily practice as rated by surgeons.

Variables	Regression coefficient (95% Confidence Interval)	Standard error	*P* value	Δ AIC*
Gender				
Women	*Reference value*			
Men	–0.24 (–0.51 to 0.023)	0.14	0.073	1.4
Practice location				
United States	*Reference value*			
Europe	0.34 (0.19 to 0.49)	0.076	**<0.001**	16
Other	0.18 (0.0075 to 0.36)	0.089	**0.041**	
Years in practice				
0 to 5	*Reference value*			
6 to 10	–0.15 (–0.32 to 0.018)	0.086	0.081	–1.4
11 to 20	–0.028 (–0.18 to 0.13)	0.080	0.721	
21 to 30	–0.12 (–0.30 to 0.051)	0.089	0.167	
Subspecialty				
Hand and wrist	*Reference value*			
Shoulder and elbow	–0.40 (–0.58 to –0.22)	0.092	**<0.001**	19
Trauma	–0.28 (–0.43 to –0.13)	0.078	**<0.001**	
Other	–0.033 (–0.22 to 0.15)	0.094	0.727	
Supervising trainees				
No	*Reference value*			
Yes	0.088 (–0.072 to 0.25)	0.082	0.281	–0.85

**Bold** indicates statistical significance, *P* < 0.05. *Δ AIC = Akaike Information Criterion, based on a single-variable negative binomial regression model. Model with best fit is used as reference value.

In qualitative analysis of surgeon comments on potential assistance, they requested 1) additional training in effective communication strategies, 2) improved practice logistics to be better prepared for and have more time available for complex patients, 3) utilization of a team-based approach, including mental and social health experts, 4) utilization of incremental care (spreading care out over several visits or touch points), 5) a more complete set of medical records in advance of the visit, 6) improve ability to discern and address mental and social health opportunities, and 7) improved stress-management and self-awareness. Conversely, several surgeons commented that they felt they would not benefit from additional training.

## 4 Discussion and conclusion

### 4.1 Discussion

In this cross-sectional survey-based experiment, we identified factors associated with surgeon-rated feelings of stress, futility, inadequacy, and frustration in office visit scenarios: multiple specialist evaluation, disproportionate symptoms, and ongoing legal dispute. Many surgeons desired additional training in effective communication strategies, discernment of the psychosocial aspects of illness, assistance with them in the office, and more effective planning and strategizing for patient encounters.

This study had several limitations. First, surgeons were asked to imagine treating these patients, which is different from seeing patients during an actual encounter. However, most surgeons in our database had multiple years of experience, seemed familiar with the scenarios modeled, and likely answered in a representative manner. Second, surgeons outside of the United States may have less experience with patients involved in civil lawsuits or Workers’ Compensation disputes. This may to some extent explain the variation in stress, frustration, etc. found between surgeons from different continents. Finally, the SOVG consists largely of white men in academic practice, which does not accurately represent all practicing surgeons. However, there was sufficient diversity of opinion in our participants that the observed associations are likely comparable across different populations of surgeons, although they might vary in magnitude. We would like to correct this lack of diversity and we welcome new members (https://www.surveymonkey.com/r/SOVGsignup).

The finding that multiple prior specialty consultations, the presence of an ongoing legal dispute, and disproportionate symptom intensity—factors that seem to reflect a mismatch between patient and specialist symptom interpretation and goals of treatment—were associated with greater physician-reported levels of stress suggests that surgeon stress may be an indicator of patient mental and social health opportunities. If surgeons can limit their stress—which is associated with lower patient-rated clinician empathy and satisfaction [21]—they may be able to foster effective relationships that can help make mental and social health a priority. Unhelpful thoughts (e.g., interpreting pain as harm), symptoms of worry and despair [22,23], and stress (e.g., job, role, and financial insecurity) [24,25] are all associated with greater symptom intensity and magnitude of incapability. Further, these factors may also be associated with mismatch between patient and specialist interpretation of symptoms. It is a normal human tendency to feel protective and prepare for the worst when feeling harmed or injured. This systematic mental tendency (cognitive bias) can present as fear of movement (kinesiophobia) or worst-case thinking [[26], [27], [28], [29]]. Such protective behaviors may be reinforced by psychological distress [22], or when people derive benefits from being ill (secondary gain), for example, when illness supports a legal dispute or garners attention [30,31]. Unhealthy thinking delays and limits recovery and is associated with greater intensity of symptoms and greater magnitude of activity intolerance [[32], [33], [34]]. Despite the growing awareness among physicians of the importance of mood and mindset on recovery [[35], [36], [37]], most surgeons receive little or no formal training in how to gently reorient misconceptions (cognitive errors). Such reorientation is difficult and may often be avoided by specialists or resisted by patients. Done poorly, this strategy may elicit a variety of negative feelings from a patient such as feeling ignored, belittled, or affronted. Surgeon training and practice in effective communication strategies has the potential to benefit both surgeon and patient well-being [[38], [39], [40], [41]]. It is more difficult to be aware of stress and use effective communication and other strategies to manage it when a surgeon feels behind in the office, as observed in our models. Future studies may further explore how clinician-experience relates to patient-reported experience (satisfaction, trust, empathy, and communication effectiveness) and identify interventions to improve the clinician experience.

The finding that similar factors (secondary gain, disproportionate symptoms, and time pressure) were associated with surgeon-rated feelings of frustration, inadequacy, and futility suggests these feelings may be correlated. In an unplanned analysis, all factors were correlated (spearman rho > 0.43, p<0.001), with frustration and stress demonstrating the strongest association (rho=0.73, p<0.001). Greater attention to surgeon experience of stress and strategies to mitigate these feelings has the potential to improve and maintain joy in practice (limited symptoms of burnout) [18].

The observation that a lower surgeon-rated proportion of interactions with notable experience of stress was associated with practicing in the US and the subspecialties ‘Shoulder and elbow’ and ‘trauma’ might reflect slight variations in the prevalence of the identified patient factors associated with stress. It might also relate to unmeasured surgeon factors (e.g. the characteristics of people that choose a specific specialty) [42], or variations in symptoms of burnout [43]. The estimated percentage of encounters with notable feelings of stress (20%) described by the participants was slightly higher than the percentage indicated by primary care physicians (15-17%) [1,44].

A noteworthy proportion of surgeons who participated in our experiment recognized the importance of discernment of the psychological aspects of the illness and effective communication strategies [12]. The fact that that many surgeons reported that they felt they would not benefit from additional training may indicate sufficient training and experience with psychosocially complex patients among practicing surgeons. On the other hand, it might represent unconscious incompetence, meaning that surgeons may be unaware of the set of skills that they lack and that would be helpful in limiting the experience of stress with patients. The sparse training in communications strategies in surgical residency training, combined with the evidence that they are important [38], may point to the latter.

### 4.2 Innovation

Immersion in the biopsychosocial paradigm, which recognizes the importance of mood and coping strategies on illness severity, learning to recognize verbal and nonverbal signs of stress and less effective pain coping strategies, practicing compassionate and effective communication strategies (e.g. motivational interviewing to help people gain awareness of their values [what matters most to them]), and improved coordination of care with social workers or clinical psychologists may help limit surgeon stress when encountering patients with notable psychosocial aspects of illness. The interventions suggested by surgeons (e.g. communication training, incremental care, use of a team-based approach) have the potential to improve the patient-clinician relationship, clinician experience and joy in practice, and patient experience including feeling trust, which could help surgeons be open to more comprehensive treatment approaches.

### 4.3 Conclusion

This survey-based experiment among an international group of practicing surgeons identified specific patient factors that may be indicative of mental and social health opportunities (e.g. ongoing legal disputes, disproportionate symptoms) associated with greater surgeon-rated stress. Surgeon stress can be anticipated, recognized and addressed with future studies required to identify which of the interventions suggested by surgeons can positively impact the clinician experience, improve job satisfaction, and increase patient satisfaction.

## Declaration of Competing Interest

One of the authors (DR) received royalties from Skeletal Dynamics for an internal joint stabilizer elbow in the amount of between 10,000 and 100,000 USD per year. One of the authors certifies that he (DR) is a Deputy Editor for Hand and Wrist, Journal of Orthopaedic Trauma, and Clinical Orthopaedics and Related Research® and has received or may receive payments or benefits in the amount of USD 5000 per year. One of the authors certifies that he (DR) received honoraria from meetings of the AO North America (Wayne, PA, USA), AO International (Davos, Switzerland), and various hospitals and universities.
